# Supplementary material for: Identifying hopelessness in population research: a validation study of two brief measures of hopelessness
Source: BMJ Open. 2014 May 30;4(5):e005093. doi: 10.1136/bmjopen-2014-005093 (PMC4039863; doi:10.1136/bmjopen-2014-005093)
Supplement: Author's manuscript [file bmjopen-2014-005093.draft_revisions.pdf]

Identifying hopelessness in population research: a validation study of two brief measures of hopelessness in a cohort of post-menopausal women in the UK.

Journal:	*BMJ Open*
Manuscript ID:	bmjopen-2014-005093
Article Type:	Research
Date Submitted by the Author:	19-Feb-2014

Complete List of Authors:	Fraser, Lindsay; UCL Institute for Women's Health and NIHR University College London Hospitals Biomedical Research Centre, Department of Women's Cancer<br>Burnell, Matthew; UCL Institute for Women's Health and NIHR University College London Hospitals Biomedical Research Centre, Department of Women's Cancer<br>Salter, Laura; University College London Hospitals NHS Foundation Trust,<br>Fourkala, Evangelia-Ourania; UCL Institute for Women's Health and NIHR University College London Hospitals Biomedical Research Centre, Department of Women's Cancer<br>Kalsi, Jatinderpal; UCL Institute for Women's Health and NIHR University College London Hospitals Biomedical Research Centre, Department of Women's Cancer<br>Ryan, Andy; UCL Institute for Women's Health and NIHR University College London Hospitals Biomedical Research Centre, Department of Women's Cancer<br>Gessler, Sue; University College London Hospitals NHS Foundation Trust, ; UCL Institute for Women's Health and NIHR University College London Hospitals Biomedical Research Centre, Department of Women's Cancer<br>Gidron, Yori; Vrije Universiteit Brussel (VUB), Faculty of Medicine and Pharmacy<br>Steptoe, Andrew; Institute of Epidemiology and Health Care, University College London<br>Menon, Usha; UCL Institute for Women's Health and NIHR University College London Hospitals Biomedical Research Centre, Department of Women's Cancer

**Primary Subject Heading**:	Epidemiology
Secondary Subject Heading:	Mental health
Keywords:	EPIDEMIOLOGY, MENTAL HEALTH, Depression & mood disorders < PSYCHIATRY

SCHOLARONE™ Manuscripts

For peer review only

TITLE

Identifying hopelessness in population research: a validation study of two brief measures of hopelessness in a cohort of post-menopausal women in the UK.

Lindsay Fraser,[1] Matthew Burnell,[1] Laura Currin Salter,[2] Evangelia-Ourania Fourkala,[1] Jatinderpal Kalsi,[1] Andy Ryan,[1] Sue Gessler,[2,1] Yori Gidron,[3] Andrew Steptoe,[4] Usha Menon[1]

Corresponding author

Ms Lindsay Fraser, Gynaecological Cancer Research Centre, Department of Women's Cancer, UCL Institute for Women's Health, Maple House, 149 Tottenham Court Road, London W1T 7DN, United Kingdom

Email: l.fraser@ucl.ac.uk

Tel: +44 020 3447 2114

Fax: +44 020 3447 2129

Author Affiliations

[1]Department of Women's Cancer, UCL Institute for Women's Health and NIHR University College London Hospitals Biomedical Research Centre, London, UK.

[2]University College London Hospitals NHS Foundation Trust, London, UK.

[3]Faculty of Medicine & Pharmacy, Vrije Universiteit Brussel (VUB), Belgium.

[4]Institute of Epidemiology and Health Care, University College London, London, UK.

Key Words: Hopelessness, Depression/epidemiology, Adult, Cohort studies.

Word Count: 2547 (Abstract=290, Manuscript=1523, References=619, Tables=115).

For peer review only

ABSTRACT

Objective: Hopelessness is an important construct in psychosocial epidemiology, but there is great pressure on the length of questionnaire measures in large scale population and clinical studies. We examined the validity and test-retest reliability of two brief measures of hopelessness, an existing negatively worded 2-item measure of hopelessness (*Brief-H-Neg*) and a positively worded version of the same instrument (*Brief-H-Pos*).

Design: Cohort study.

Setting: Control arm of the UK Collaborative Trial of Ovarian Cancer Screening.

Participants: A non-clinical research-based sample of 5000 postmenopausal women selected from 56512 participants.

Primary and secondary outcome measures: Spearman rank correlation of brief measures of hopelessness with Beck Hopelessness Scale (*BHS*). Spearman rank correlation with Centre for Epidemiological Studies Depression Scale (*CES-D*) and change in mean score on repeat testing.

Methods: Two short hopelessness measures – a negatively worded brief measure of hopelessness (*Brief-H-Neg*) and positively worded brief measure of hopelessness (*Brief-H-Pos*) were administered by postal questionnaire to 5000 women together with the 20-item Beck Hopelessness Scale (*BHS*) and 20-item Centre for Epidemiologic Studies Depression scale (*CES-D*). The *Brief-H-Neg* and *Brief-H-Pos* were re-administered to 500 women after a two week interval.

Results: 2413 postmenopausal women (mean age 68.9 years) completed the questionnaire. The *Brief-H-Neg* and *Brief-H-Pos* correlated 0.93 and 0.87 with the *BHS* after correction for attenuation and their association with the *CES-D* mirrored that seen with the BHS (Spearman rank correlation 0.88 and 0.68 respectively). There was no change in mean scores on the two measures with repeat testing in the 433 women who completed it and test-retest reliability was good (Intraclass correlations *Brief-H-Neg* 0.67 and *Brief-H-Pos* 0.72).

For peer review only

Conclusions: These findings provide support for the validity of the *Brief-H-Neg* and *Brief-H-Pos*. These brief measures are likely to be useful in large population studies assessing hopelessness.

ARTICLE SUMMARY

Article Focus

- Research into the role of hopelessness on morbidity and mortality is hampered by the lack of validated brief measures for use in large scale clinical and population studies.
- This study explored the validity and reliability of an existing brief measure of hopelessness with negatively worded items, along with a newly created measure with positively worded items which may be preferred in some research settings.

Key Messages

- The results show that both the original negatively worded brief instrument and the new positively worded version are valid and reliable instruments for measuring hopelessness.
- It is recognised that brief measures of hopelessness necessarily sacrifice some level of accuracy compared to longer measures, but are useful when there is great pressure on questionnaire length.
- While the predictive validity of the original negatively worded measure has previously been demonstrated, this remains to be tested in the positively worded measure.

Strengths and Limitations

- The strength of this study is the large sample size.
- It is not known whether the positively worded measure of hopelessness is associated with less participant distress compared to the negatively worded measure, and this should be addressed.

For peer review only

INTRODUCTION

Hopelessness is the subjective appraisal of negative expectations about the occurrence of highly valued outcomes coupled with the sense that one lacks control over desired events in the future.[1] Hopelessness has been related to the onset and prognosis of mental and physical health outcomes including the development of depression,[1] hypertension,[2] subclinical atherosclerosis,[3, 4] adaptation following acute cardiac events[5] and progression of carotid atherosclerosis.[3] In the psycho-oncology literature hopelessness has been found to predict prognosis in various cancers including breast and haematological cancers,[6, 7] although the evidence is not consistent.

Hopelessness has been measured in clinical and population research in a variety of ways including systematic interviews[8] and validated psychometric measures such as the Beck Hopelessness Scale[9] and the Mental Adjustment to Cancer scale.[10] There is great pressure in large scale population studies on questionnaire size due to the volume of clinical and demographic variables that must be collected. Everson et al[11] devised a 2-item measure of hopelessness which has been used in a number of cardiovascular studies.[2, 3, 11] The reliability of this instrument and its relationship with standard measures has not been established. An additional issue concerns the negative valence of the items (e.g. 'The future seems to me to be hopeless and I can't believe that things are changing for the better'). In preliminary work for the large study in which this research is embedded, some respondents found these items upsetting and this has been confirmed by others.[12] We devised a positively worded 2-item version. We compared both brief measures with established measures of hopelessness and depressive symptoms in a large population sample, and assessed their reliability.

METHODS

Participants

5000 participants were selected from 56512 post-menopausal women in the control arm of the UK Collaborative Trial of Ovarian Cancer Screening (UKCTOCS,[13]

For peer review only - http://bmjopen.bmj.com/site/about/guidelines.xhtml

ISRCTN22488978, ClinicalTrials.gov number NCT00058032). The mean age of women invited was 69.6 +/- 6.1 years (range: 57-85).

Procedure

A postal questionnaire comprised of measures of hopelessness and depression was sent to 5000 women (Time 1, T1). After a 2-week interval (Time 2, T2) 500 respondents were asked to repeat the *Brief-H-Neg* (n=250) or the *Brief-H-Pos* (n=250) to assess test-retest reliability. Selection of the retest cohort was staggered based on the date of T1 questionnaire return, as early and late responders may differ on levels of hopelessness or depression.[14]

Measures

The *Brief-H-Neg* is a 2-item measure of hopelessness comprised of negatively valenced statements: 'The future seems to me to be hopeless and I can't believe that things are changing for the better'; 'I feel that it is impossible to reach the goals I would like to strive for'.[11] Everson et al selected these from a battery of psychosocial measures used in the Kuopio Ischemic Heart Disease study, defining hopelessness as negative expectancies about oneself and the future. Respondents indicate agreement on a 5-point scale (range: 2-10), higher scores indicate higher hopelessness.

The *Brief-H-Pos* was derived by reversing the tone of the *Brief-H-Neg* statements from negative to positive and reverse scoring: 'The future seems to me to be hopeful and I believe that things are changing for the better'; 'I feel that it is possible to reach the goals I would like to strive for'.

The *BHS* is a validated 20-item true-false measure assessing current levels of hopelessness.[9] Items include pessimistic statements ('There's no use in really trying to get something I want because I probably won't get it') and optimistic ones ('I look forward to the future with hope and enthusiasm'). Pessimistic ratings are summed (range: 0-20), higher scores indicate higher hopelessness.

The Centre for Epidemiological Studies Depression Scale (*CES-D*) is a validated 20-item measure of depressive symptoms.[15] Responses are based on the frequency of

For peer review only

occurrence during the past week using a 4-point scale (range: 0-60), higher scores indicate more frequent symptoms of depression.

Analyses

Internal consistency was based on Coefficient Alpha[16] with alpha cut-off points 0.70-0.79 described as *adequate* and ≥0.80 as high.[17] Stability was evaluated using test-retest reliability based on the Intraclass Correlation Coefficient (ICC) with cut-offs ≤0.40 for *poor*, 0.41-0.59 *fair*, 0.60-0.74 *good*, ≥0.75 excellent.[18] Estimated variances components derived from a one-way random effects model were used to calculate ICC's.[19] The relationship between study measures was assessed using Spearman's rank correlations (CIs were estimated using bootstrapping with 1000 iterations).[20] To estimate the strength of correlations between study measures, a correction for attenuation arising from measurement error was applied: $\rho_{xy} = r_{xy}$/*square root symbol* ($r_{xx}.r_{yy}$),[21, 22] where ρ_{xy} = true correlation between x and y, r_{xy} = observed correlation between x and y, r_{xx} = estimated reliability of x, r_{yy} = estimated reliability of y. We used published test-retest reliability estimates for r_{xx} and r_{yy}: *BHS* 0.69[23] and *CES-D* 0.67.[15] In the absence of published test-retest data for the *Brief-H-Neg*/*Brief-H-Pos*, we used the ICCs reported in this study. Data were analysed using STATA, Version 12.1.[24]

RESULTS

Sample Characteristics

The questionnaire was returned by 2413 women (48.3%) (T1) (Table 1). Respondents reported significantly higher levels of education than non-respondents, were younger and more likely to be Caucasian (differences were not clinically significant, due to their small magnitude). 115 respondents (4.77%) scored CES-D ≥16/60, a cut-off indicative of clinically significant depressive symptomatology, suggesting this cohort is not unusually depressed.

For peer review only

Table 1 Description of respondents' characteristics

	**Respondents** (N=2413)
**Age** in years (mean +/- SD)	68.9 +/- 5.9 (range: 57-84)
**Ethnicity** n (%)	
White	2376 (98.7)
Black	11 (0.5)
Asian	7 (0.3)
Other	14 (0.6)
Unknown	5 (0.2)
**Education** n (%)	
Higher (University, Professional)	819 (33.9)
Some (O' Level, A' Level, Clerical)	955 (39.6)
None	610 (25.3)
Unknown	29 (1.2)
**Hopelessness** (mean +/- SD)	
Brief-H-Neg	4.42 +/- 2.21 (n=2402)
Brief-H-Pos	4.74 +/- 1.85 (n=2393)
BHS	4.81 +/- 4.49 (n=2400)
**Depression** (mean +/- SD)	
CES-D	12.44 +/- 10.39 (n=2395)

For peer review only

Construct Validity

The *Brief-H-Neg* and *Brief-H-Pos* measures correlated well with the *BHS* and mirrored the association seen between the *BHS* and the *CES-D* (Table 2).

Table 2 Correlation between measures of hopelessness and depression

	Brief-H-Neg (n)	Brief-H-Pos (n)	BHS (n)
**BHS**	0.93 (2393)	0.87 (2384)	
**CES-D**	0.88 (2379)	0.68 (2392)	0.87 (2379)

Stability

433/497 (87.1%) women completed the *Brief-H-Neg* (n=221) or *Brief-H-Pos* (n=212) on two occasions. *Brief-H-Neg*, T1 M = 4.64 +/- 1.74 (*n*=248), T2 M = 4.29 +/- 2.39 (*n*=221); *Brief-H-Pos*, T1 M = 4.61 +/- 1.878 (*n*=249), T2 M = 4.57 +/- 1.96 (*n*=212). The short term test-retest reliability of both measures was good: *Brief-H-Neg* ICC = 0.67 (95% CI 3.98-4.49) and *Brief-H-Pos* ICC = 0.72 (95% CI 4.39-4.83).

Reliability

All study measures demonstrated good internal consistency: *Brief-H-Neg* α 0.80, *Brief-H-Pos* α 0.77, *BHS* α 0.89, *CES-D* α 0.90. Alpha for the *Brief-H-Neg* and *Brief-H-Pos* was lower than the longer BHS and CES-D (alpha is known to rise as the number of items increase).

DISCUSSION

A brief measure is needed to examine the role of hopelessness on mental and physical health outcomes in large population studies. We examined the validity and reliability of two brief measures of hopelessness in a large non-clinical sample, one negatively valenced (*Brief-H-Neg*) and one positively valenced (*Brief-H-Pos*). Both were shown to have good construct validity, correlating strongly with the longer BHS and mirroring

For peer review only

the association seen between the *BHS* and a measure of depression, and adequate test-retest reliability and internal consistency.

Two-item measures of psychological constructs are used when very short measures are needed.[25, 26] Although two questions may seem a small amount of information to base a judgement of hopelessness on, the 5-point response scale provides a reasonable range of scores to work with. Very brief measures necessarily sacrifice some level of accuracy for efficiency compared to their longer counterparts.[27]

We did not test the assumption that those suffering from low mood may find it difficult to be confronted with negatively phrased questions and this should be addressed. The predictive validity for the *Brief-H-Neg* has been shown in studies exploring the relationship between hopelessness and disease incidence and mortality.[2-4, 11] The predictive validity of the *Brief-H-Pos* needs to be assessed.

CONCLUSION

Everson et al's negatively valenced measure of hopelessness and our positively valenced measure developed as a potentially less stressful measure for participants in health research have been shown to be valid and reliable measures of hopelessness. These brief measures are likely to be useful in large scale population studies investigating the role of hopelessness in health outcomes when questionnaire length is constrained.

Contributors

All authors contributed to the study design. JK and AR collected the data. MB conducted the data analysis. MB, LF, UM and AS interpreted the data. LF, UM and AS drafted the manuscript. All authors revised and approved the final version of the manuscript.

Funding

UKCTOCS is core funded by the Medical Research Council (G0801228), Cancer Research UK (C1479/A2884), The Department of Health, with additional support from The Eve Appeal, Special Trustees of Barts and the London, Special Trustees of UCLH. The funding bodies had no role in study design, data collection, analysis, interpretation or writing of this report.

For peer review only

Competing Interests

The authors have no competing interests to report.

Patient Consent

Obtained.

Ethics Approval

Ethical approval was obtained from the NRES Committee North West-Haydock (00/8/034).

Provenance and Peer Review

Not commissioned; externally peer reviewed.

Data Sharing Agreement

The data from the current study are available to specific researchers at the Gynaecological Cancer Research Centre, UCL Institute for Women's Health.

Open Access

This is an Open Access article distributed in accordance with the Creative Commons Attribution Non Commercial (CC BY-NC 3.0) license, which permits others to distribute, remix, adapt, build upon this work non-commercially, and license their derivative works on different terms, provided the original work is properly cited and the use is non-commercial. See: http://creativecommons.org/licenses/by-nc/3.0/

REFERENCES

[1.] Alloy LB, Abramson LY, Metalsky GI, et al. The hopelessness theory of depression: attributional aspects. *Br J Clin Psychol* 1988;**27**:5-21.

[2.] Everson SA, Kaplan GA, Goldberg DE, et al. Hypertension incidence is predicted by high levels of hopelessness in Finnish men. *Hypertension* 2000;**35**:561-7.

[3.] Everson SA, Kaplan GA, Goldberg DE, et al. Hopelessness and 4-year progression of carotid atherosclerosis. The Kuopio Ischemic Heart Disease Risk Factor Study. *Arterioscler Thromb Vasc Biol* 1997;**17**:1490-5.

[4.] Whipple MO, Lewis TT, Sutton-Tyrrell K, et al. Hopelessness, depressive symptoms, and carotid atherosclerosis in women: the Study of Women's Health

For peer review only

Across the Nation (SWAN) heart study. *Stroke* 2009;**40**:3166-72. doi: 10.1161/STROKEAHA.109.554519.

[5.] Dunn SL, Corser W, Stommel M, et al. Hopelessness and depression in the early recovery period after hospitalization for acute coronary syndrome. *J Cardiopulm Rehabil* 2006;**26**:152-9.

[6.] Watson M, Homewood J, Haviland J. Coping response and survival in breast cancer patients: a new analysis. *Stress Health* 2012;**28**:376-80. doi: 10.1002/smi.2459.

[7.] Molassiotis A, Van Den Akker OB, Milligan DW, et al. Symptom distress, coping style and biological variables as predictors of survival after bone marrow transplantation. *J Psychosom Res* 1997;**42**:275-85.

[8.] Morris TP, K.; Haybittle, J. Psychological response to cancer diagnosis and disease outcome in patients with breast cancer and lymphoma. *Psychooncology* 1992;**1**:105-14.

[9.] Beck AT, Weissman A, Lester D, et al. The measurement of pessimism: the hopelessness scale. *J Consult Clin Psychol* 1974;**42**:861-5.

[10.] Watson M, Haviland JS, Greer S, et al. Influence of psychological response on survival in breast cancer: a population-based cohort study. *Lancet* 1999;**354**:1331-6.

[11.] Everson SA, Goldberg DE, Kaplan GA, et al. Hopelessness and risk of mortality and incidence of myocardial infarction and cancer. *Psychosom Med* 1996;**58**:113-21.

[12.] Kohout FJ, Berkman LF, Evans DA, et al. Two shorter forms of the CES-D (Center for Epidemiological Studies Depression) depression symptoms index. *J Aging Health* 1993;**5**:179-93.

[13.] Menon U, Gentry-Maharaj A, Hallett R, et al. Sensitivity and specificity of multimodal and ultrasound screening for ovarian cancer, and stage distribution of detected cancers: results of the prevalence screen of the UK Collaborative Trial of

For peer review only

Ovarian Cancer Screening (UKCTOCS). *Lancet Oncol* 2009;**10**:327-40. doi: 10.1016/S1470-2045(09)70026-9.

[14.] Leadbetter S, Hawkins NA, Scholl LE, et al. Recruiting women for a study on perceived risk of cancer: influence of survey topic salience and early versus late response. Prev Chronic Dis 2013;**10**:E75. doi: 10.5888/pcd10.120293.

[15.] Radloff LS. The CES-D scale: A self-report depression scale for research in the general population. *Appl Psychol Meas* 1977;**1**:385-401.

[16.] Cronbach LJ. Coefficient alpha and the internal structure of tests. *Psychometrika* 1951;**16**:297-334.

[17.] Streiner DL. Starting at the beginning: an introduction to coefficient alpha and internal consistency. *J Pers Assess* 2003;**80**:99-103. doi: 10.1207/S15327752JPA8001_18.

[18.] Cicchetti DV. Multiple comparison methods: establishing guidelines for their valid application in neuropsychological research. *J Clin Exp Neuropsychol* 1994;**16**:155-61.

[19.] Shrout PE, Fleiss JL. Intraclass correlations: uses in assessing rater reliability. *Psychol Bull* 1979;**86**:420-8.

[20.] Haukoos JS, Lewis RJ. Advanced statistics: bootstrapping confidence intervals for statistics with "difficult" distributions. *Acad Emerg Med* 2005;**12**:360-5. doi: 10.1197/j.aem.2004.11.018.

[21.] Krueger AB, Schkade DA. The Reliability of Subjective Well-Being Measures. *J Public Econ* 2008;**92**:1833-45. doi: 10.1016/j.jpubeco.2007.12.015.

[22.] Nunnally JC. *Psychometric Theory.* 2nd ed. New York: McGraw Hill, 1978.

[23.] Aiken LR. *Psychological testing and assessment.* 11th ed. Boston: Allyn & Bacon, 2003.

[24.] Stata Statistical Software: Release 12. College Station, Texas: StataCorp LP, 2011.

For peer review only

[25.] Gosling SD, Rentfrow PJ, Swann, WB. A very brief measure of the Big-Five personality domains. *J Res Pers* 2003;**37**:504-28.

[26.] Weinstein N, Brown KW, Ryan, RM. A multi-method examination of the effects of mindfulness on stress attribution, coping, and emotional well-being. *J Res Pers* 2009;**43**:374-85.

[27.] Jonason PK, Luevano VX. Walking the thin line between efficiency and accuracy: Validity and structural properties of the Dirty Dozen. *Pers Individ Dif* 2013;**5**:76-81.

For peer review only

BMJ Open

Identifying hopelessness in population research: a validation study of two brief measures of hopelessness.

Journal:	*BMJ Open*
Manuscript ID:	bmjopen-2014-005093.R1
Article Type:	Research
Date Submitted by the Author:	28-Apr-2014
Complete List of Authors:	Fraser, Lindsay; UCL Institute for Women's Health and NIHR University College London Hospitals Biomedical Research Centre, Department of Women's Cancer<br>Burnell, Matthew; UCL Institute for Women's Health and NIHR University College London Hospitals Biomedical Research Centre, Department of Women's Cancer<br>Salter, Laura; University College London Hospitals NHS Foundation Trust,<br>Fourkala, Evangelia-Ourania; UCL Institute for Women's Health and NIHR University College London Hospitals Biomedical Research Centre, Department of Women's Cancer<br>Kalsi, Jatinderpal; UCL Institute for Women's Health and NIHR University College London Hospitals Biomedical Research Centre, Department of Women's Cancer<br>Ryan, Andy; UCL Institute for Women's Health and NIHR University College London Hospitals Biomedical Research Centre, Department of Women's Cancer<br>Gessler, Sue; University College London Hospitals NHS Foundation Trust, ; UCL Institute for Women's Health and NIHR University College London Hospitals Biomedical Research Centre, Department of Women's Cancer<br>Gidron, Yori; Vrije Universiteit Brussel (VUB), Faculty of Medicine and Pharmacy<br>Steptoe, Andrew; Institute of Epidemiology and Health Care, University College London<br>Menon, Usha; UCL Institute for Women's Health and NIHR University College London Hospitals Biomedical Research Centre, Department of Women's Cancer
**Primary Subject Heading**:	Epidemiology
Secondary Subject Heading:	Mental health
Keywords:	EPIDEMIOLOGY, MENTAL HEALTH, Depression & mood disorders < PSYCHIATRY

SCHOLARONE™
Manuscripts

TITLE

Identifying hopelessness in population research: A validation study of two brief measures of hopelessness.

Lindsay Fraser,[1] Matthew Burnell,[1] Laura Currin Salter,[2] Evangelia-Ourania Fourkala,[1] Jatinderpal Kalsi,[1] Andy Ryan,[1] Sue Gessler,[2,1] Yori Gidron,[3] Andrew Steptoe,[4] Usha Menon[1]

Corresponding author

Ms Lindsay Fraser, Gynaecological Cancer Research Centre, Department of Women's Cancer, UCL Institute for Women's Health, Maple House, 149 Tottenham Court Road, London W1T 7DN, United Kingdom

Email: l.fraser@ucl.ac.uk

Tel: +44 020 3447 2114

Fax: +44 020 3447 2129

Author Affiliations

[1]Department of Women's Cancer, UCL Institute for Women's Health and NIHR University College London Hospitals Biomedical Research Centre, London, UK.

[2]University College London Hospitals NHS Foundation Trust, London, UK.

[3]Faculty of Medicine & Pharmacy, Vrije Universiteit Brussel (VUB), Belgium.

[4]Institute of Epidemiology and Health Care, University College London, London, UK.

Key Words: Hopelessness, Depression/epidemiology, Adult, Cohort studies.

Word Count: 3308 (Abstract=289, Manuscript=2,116, References=860, Tables=114).

For peer review only

ABSTRACT

Objective: Hopelessness is an important construct in psychosocial epidemiology, but there is great pressure on the length of questionnaire measures in large scale population and clinical studies. We examined the validity and test-retest reliability of two brief measures of hopelessness, an existing negatively worded 2-item measure of hopelessness (*Brief-H-Neg*) and a positively worded version of the same instrument (*Brief-H-Pos*).

Design: Cohort study.

Setting: Control arm of the UK Collaborative Trial of Ovarian Cancer Screening.

Participants: A non-clinical research-based sample of 5000 postmenopausal women selected from 56512 participants.

Primary and secondary outcome measures: Spearman rank correlation of brief measures of hopelessness with Beck Hopelessness Scale (*BHS*). Spearman rank correlation with Centre for Epidemiological Studies Depression Scale (*CES-D*) and change in mean score on repeat testing.

Methods: Two short hopelessness measures, a negatively worded brief measure of hopelessness (*Brief-H-Neg*) and positively worded brief measure of hopelessness (*Brief-H-Pos*), were administered by postal questionnaire to 5000 women together with the 20-item Beck Hopelessness Scale (*BHS*) and 20-item Centre for Epidemiologic Studies Depression scale (*CES-D*). The *Brief-H-Neg* and *Brief-H-Pos* were re-administered to 500 women after a two week interval.

Results: 2413 postmenopausal women (mean age 68.9 years) completed the questionnaire. The *Brief-H-Neg* and *Brief-H-Pos* correlated 0.93 and 0.87 with the *BHS* after correction for attenuation and their association with the *CES-D* mirrored that seen with the BHS (Spearman rank correlation 0.88 and 0.68 respectively). There was no change in mean scores on the two measures with repeat testing in the 433 women who completed it and test-retest reliability was good (Intraclass correlations *Brief-H-Neg* 0.67 and *Brief-H-Pos* 0.72).

For peer review only

Conclusions: These findings provide support for the validity of the *Brief-H-Neg* and *Brief-H-Pos*. These brief measures are likely to be useful in large population studies assessing hopelessness.

ARTICLE SUMMARY

Article Focus

- Research into the role of hopelessness on morbidity and mortality is hampered by the lack of validated brief measures for use in large scale clinical and population studies.
- This study explored the validity and reliability of an existing brief measure of hopelessness with negatively worded items, along with a newly created measure with positively worded items which may be preferred in some research settings.

Key Messages

- Both the original negatively worded brief instrument and the new positively worded version are valid and reliable instruments for measuring hopelessness.
- While brief measures of hopelessness necessarily sacrifice some level of detail compared to longer measures, they could be useful when there is great pressure on questionnaire length.
- The predictive validity of the original negatively worded measure has previously been demonstrated and this remains to be tested in the positively worded measure.
- Further testing to verify the construct validity of the two brief measures is warranted.

Strengths and Limitations

- The strength of this study is the large sample size.
- Limitations include generalizability of the results beyond older women and the modest response rate.

For peer review only

INTRODUCTION

Hopelessness is the subjective appraisal of negative expectations about the occurrence of highly valued outcomes coupled with the sense that one lacks control over desired events in the future.[1] Hopelessness has been related to the onset and prognosis of mental and physical health outcomes including the development of depression,[1] suicidal ideation,[2] hypertension,[3] subclinical atherosclerosis,[4, 5] adaptation following acute cardiac events[6] and progression of carotid atherosclerosis.[4] In the psycho-oncology literature hopelessness has been found to predict prognosis in various cancers including breast and haematological cancers,[7, 8] although the evidence is not consistent.

Hopelessness has been measured in clinical and population research in a variety of ways including systematic interviews[9] and validated psychometric measures such as the Beck Hopelessness Scale[10] and the Mental Adjustment to Cancer scale.[11] There is great pressure in large scale population studies on questionnaire size due to the volume of clinical and demographic variables that must be collected. Everson et al[12] devised a 2-item measure of hopelessness which has been used in a number of cardiovascular studies.[3, 4, 12] The reliability of this instrument and its relationship with standard measures has not been established. An additional issue concerns the negative valence of the items (e.g. 'The future seems to me to be hopeless and I can't believe that things are changing for the better'). In preliminary work for the large study in which this research is embedded, some respondents found these items upsetting and this has been confirmed by others.[13] We devised a positively worded 2-item version. We compared both brief measures with established measures of hopelessness and depressive symptoms in a large population sample, and assessed their reliability.

METHODS

Participants

5000 participants were selected from 56512 post-menopausal women in the control arm of the UK Collaborative Trial of Ovarian Cancer Screening (UKCTOCS,[14]

For peer review only

ISRCTN22488978, ClinicalTrials.gov number NCT00058032). The mean age of women invited was 69.6 +/- 6.1 years (range: 57-85).

Procedure

A postal questionnaire comprised of measures of hopelessness and depression was sent to 5000 women (Time 1, T1). After a 2-week interval (Time 2, T2) 500 respondents were asked to repeat the *Brief-H-Neg* (n=250) or the *Brief-H-Pos* (n=250) to assess test-retest reliability. Selection of the retest cohort was staggered based on the date of T1 questionnaire return, as early and late responders may differ on levels of hopelessness or depression.[15]

Measures

The *Brief-H-Neg* is a 2-item measure of hopelessness comprised of negatively valenced statements: 'The future seems to me to be hopeless and I can't believe that things are changing for the better'; 'I feel that it is impossible to reach the goals I would like to strive for'.[12] Everson et al selected these from a battery of psychosocial measures used in the Kuopio Ischemic Heart Disease study, defining hopelessness as negative expectancies about oneself and the future. Respondents indicate agreement on a 5-point scale (range: 2-10), higher scores indicate higher hopelessness (Appendix A).

The *Brief-H-Pos* was derived by reversing the tone of the *Brief-H-Neg* statements from negative to positive and reverse scoring: 'The future seems to me to be hopeful and I believe that things are changing for the better'; 'I feel that it is possible to reach the goals I would like to strive for' (Appendix B).

The *BHS* is a validated 20-item true-false measure assessing current levels of hopelessness.[10] Items include pessimistic statements ('There's no use in really trying to get something I want because I probably won't get it') and optimistic ones ('I look forward to the future with hope and enthusiasm'). Pessimistic ratings are summed (range: 0-20), higher scores indicate higher hopelessness.

The Centre for Epidemiological Studies Depression Scale (*CES-D*) is a validated 20-item measure of depressive symptoms.[16] Responses are based on the frequency of

For peer review only

occurrence during the past week using a 4-point scale (range: 0-60), higher scores indicate more frequent symptoms of depression.

Analyses

Internal consistency was based on Coefficient Alpha[17] with alpha cut-off points 0.70-0.79 described as *adequate* and ≥0.80 as high.[18] Stability was evaluated using test-retest reliability based on the Intraclass Correlation Coefficient (ICC) with cut-offs ≤0.40 for *poor*, 0.41-0.59 *fair*, 0.60-0.74 *good*, ≥0.75 excellent.[19] Estimated variances components derived from a one-way random effects model were used to calculate ICC's.[20] The relationship between study measures was assessed using Spearman's rank correlations (CIs were estimated using bootstrapping with 1000 iterations).[21] To estimate the strength of correlations between study measures, a correction for attenuation arising from measurement error was applied: $\rho_{xy} = r_{xy}$/*square root symbol* $(r_{xx}.r_{yy})$,[22, 23] where ρ_{xy} = true correlation between x and y, r_{xy} = observed correlation between x and y, r_{xx} = estimated reliability of x, r_{yy} = estimated reliability of y. We used published test-retest reliability estimates for r_{xx} and r_{yy}: *BHS* 0.69[24] and *CES-D* 0.67.[16] In the absence of published test-retest data for the *Brief-H-Neg*/*Brief-H-Pos*, we used the ICCs reported in this study. Data were analysed using STATA version 12.1.

RESULTS

Sample Characteristics

The questionnaire was returned by 2413 women (48.3%) (T1) (Table 1). Respondents reported significantly higher levels of education than non-respondents, were younger and more likely to be Caucasian (differences were not clinically significant, due to their small magnitude). 115 respondents (4.77%) scored CES-D ≥16/60, a cut-off indicative of clinically significant depressive symptomatology, suggesting this cohort is not unusually depressed.

Table 1 Description of respondents' characteristics

For peer review only

	**Respondents** (N=2413)
**Age** in years (mean +/- SD)	68.9 +/- 5.9 (range: 57-84)
**Ethnicity** n (%)	
White	2376 (98.7)
Black	11 (0.5)
Asian	7 (0.3)
Other	14 (0.6)
Unknown	5 (0.2)
**Education** n (%)	
Higher (University, Professional)	819 (33.9)
Some (O' Level, A' Level, Clerical)	955 (39.6)
None	610 (25.3)
Unknown	29 (1.2)
**Hopelessness** (mean +/- SD)	
Brief-H-Neg	4.42 +/- 2.21 (n=2402)
Brief-H-Pos	4.74 +/- 1.85 (n=2393)
BHS	4.81 +/- 4.49 (n=2400)
**Depression** (mean +/- SD)	
CES-D	12.44 +/- 10.39 (n=2395)

For peer review only

Concurrent Validity

The *Brief-H-Neg* and *Brief-H-Pos* measures correlated well with the *BHS* and mirrored the positive association seen between the *BHS* and the *CES-D* (Table 2).

Table 2 Correlation between measures of hopelessness and depression

	**Brief-H-Neg**	**Brief-H-Pos**	**BHS**

	(n)	(n)	(n)
**BHS**	0.93 (2393)	0.87 (2384)	
**CES-D**	0.88 (2379)	0.68 (2392)	0.87 (2379)

Stability

433/497 (87.1%) women completed the *Brief-H-Neg* (n=221) or *Brief-H-Pos* (n=212) on two occasions. *Brief-H-Neg*, T1 M = 4.64 +/- 1.74 (*n*=248), T2 M = 4.29 +/- 2.39 (*n*=221); *Brief-H-Pos*, T1 M = 4.61 +/- 1.878 (*n*=249), T2 M = 4.57 +/- 1.96 (*n*=212). The short term test-retest reliability of both measures was good: *Brief-H-Neg* ICC = 0.67 (95% CI 3.98-4.49) and *Brief-H-Pos* ICC = 0.72 (95% CI 4.39-4.83).

Reliability

All study measures demonstrated good internal consistency: *Brief-H-Neg* α 0.80, *Brief-H-Pos* α 0.77, *BHS* α 0.89, *CES-D* α 0.90. Alpha for the *Brief-H-Neg* and *Brief-H-Pos* was lower than the longer BHS and CES-D (alpha is known to rise as the number of items increase).

DISCUSSION

A brief measure is needed to examine the role of hopelessness on mental and physical health outcomes in large population studies. We examined the validity and reliability of two brief measures of hopelessness in a large non-clinical sample, one negatively valenced (*Brief-H-Neg*) and one positively valenced (*Brief-H-Pos*). Both were shown to correlate strongly with the longer BHS and mirror the positive correlation seen between the *BHS* and a measure of depression, providing evidence of concurrent validity, with adequate internal consistency and test-retest reliability.

The size of the 2-week retest correlations for the brief measures reported in our non-clinical sample (0.67 and 0.72) are similar to those reported for the BHS in a sample of

For peer review only

university undergraduates over a 3-week retest interval (0.67, female students) or a 10-week interval (0.75).[25, 26] Studies assessing retest reliability of hopelessness instruments have reported varying retest intervals. Hopelessness may be conceptualised as a temporary mood state reflecting a person's response to challenging circumstances, or a more enduring trait reflecting a habitual outlook on many aspects of life.[27] Most commonly used measures of hopelessness, including the BHS, do not distinguish between state and trait hopelessness. If hopelessness is an enduring trait, measures of hopelessness would be expected to have high test-retest reliability. A measure that does addresses the state versus trait distinction, the State-Trait Hopelessness Scale, has reported retest correlations of state and trait hopelessness over a 6-week interval (state 0.65, trait 0.74) and over a 6-month interval (state 0.61, trait 0.78) in hospitalised patients with coronary heart disease.[28] Again, the size of these retest correlations are not dissimilar to those seen in the brief measures reported in our study after a 2-week interval.

The selection of a measure is determined to an extent by the practical context of the investigation. Very brief measures necessarily sacrifice some level of detail compared with their longer counterparts.[29] A pooled analysis and meta-analysis of 22 studies involving ultra-short (one-, two-, three- or four-item) tests concluded that 2-item and 3-item measures of depression identify 8 out of 10 cases in primary care settings, albeit at the expense of a high false positive rate.[30] This makes them inappropriate diagnostic tests for clinical decision making, but suitable as screening tools in primary care as well as in population cohort research where participants have to complete a number of demographic and clinical questions in addition to psychological measures.[31]

Our data suggest that while 2-item measures of hopelessness may not have the detail of the 20-item BHS measure, they do have adequate reliability to be used in large population based studies. The reduced burden on participants may encourage a high response rate. The 5-point Likert response scales of the *Brief-H-Neg* and *Brief-H-Pos* provide a reasonable range of scores to work with. However, if information on the hypothesised affective, motivational and cognitive aspects of hopelessness is required in

For peer review only

order for example to target a therapeutic intervention, the 20-item BHS would be more suitable, because a total score for each dimension can be derived from the summed individual items of the scale.[10]

The results of this study provide preliminary support for the construct validity of both brief measures of hopelessness but further testing of their construct validity is required, along with tests of their predictive validity on physical and mental health outcomes. It would be helpful to examine the psychometric properties of both brief measures in a psychiatric sample where higher levels of hopelessness are expected, such as a group of hospitalised patients who have attempted suicide.[32] There is good evidence that hopelessness is associated with suicidal ideation and is recognised as a better predictor for suicidal intent than depression.[33] Moreover, brief measures of hopelessness derived from the BHS including a 4-item scale and to a lesser extent a single item, have been shown to perform as well as the 20-item BHS in identifying people with suicidal ideation.[34] The predictive validity for the *Brief-H-Neg* on physical health outcomes has been shown in studies exploring the relationship between hopelessness and disease incidence and mortality, and this remains to be addressed for the *Brief-H-Pos*.[3-5, 12]

There are some limitations to this study. Firstly, the sample of older women limits the generalizability of the results. It would be useful to validate the *Brief-H-Neg* and *Brief-H-Pos* in a general population sample and to generate normative data, as has been shown for 2-item measures of depression (PHQ-2) and anxiety (GAD-2).[35] Secondly, the response rate of 48.3% is modest, although importantly there was no evidence of bias between responders and non-responders and the sample of responders is large. It is perhaps unsurprising that many of the women invited from the control arm of an ovarian cancer screening study were not motivated to take part in this nested study assessing brief measures of hopelessness. Lastly, we did not directly test the assumption that those suffering from low mood may find it difficult to be confronted with the negatively phrased questions of the *Brief-H-Neg* compared with the positively phrased *Brief-H-Pos*.

For peer review only

CONCLUSION

Both Everson et al's negatively valenced measure of hopelessness (*Brief-H-Neg*) and the positively valenced measure (*Brief-H-Pos*) developed as a potentially less stressful measure for participants in health research have been shown to be valid and reliable measures of hopelessness. Further testing to verify their construct validity is warranted. Meanwhile the findings suggest that these brief measures are fit for purpose in large scale population studies investigating the association of hopelessness and health outcomes. Evidence of a consistent association with mortality in such studies would add impetus to the search for interventions that can modify the risk.

Contributors

All authors contributed to the study design. JK and AR collected the data. MB conducted the data analysis. MB, LF, UM and AS interpreted the data. LF, UM and AS drafted the manuscript. All authors revised and approved the final version of the manuscript.

Funding

UKCTOCS is core funded by the Medical Research Council (G0801228), Cancer Research UK (C1479/A2884), The Department of Health, with additional support from The Eve Appeal, Special Trustees of Barts and the London, Special Trustees of UCLH. The funding bodies had no role in study design, data collection, analysis, interpretation or writing of this report.

Competing Interests

The authors have no competing interests to report.

Patient Consent

Obtained.

Ethics Approval

Ethical approval was obtained from the NRES Committee North West-Haydock (00/8/034).

Provenance and Peer Review

Not commissioned; externally peer reviewed.

Data Sharing Agreement

For peer review only

The data from the current study are available to specific researchers at the Gynaecological Cancer Research Centre, UCL Institute for Women's Health.

Open Access

This is an Open Access article distributed in accordance with the Creative Commons Attribution Non Commercial (CC BY-NC 3.0) license, which permits others to distribute, remix, adapt, build upon this work non-commercially, and license their derivative works on different terms, provided the original work is properly cited and the use is non-commercial. See: http://creativecommons.org/licenses/by-nc/3.0/

REFERENCES

1. Alloy LB, Abramson LY, Metalsky GI, *et al*. The hopelessness theory of depression: attributional aspects. *Br J Clin Psychol* 1988;27(Pt 1):5-21.
2. Beck AT, Steer RA, Kovacs M, *et al*. Hopelessness and eventual suicide: a 10-year prospective study of patients hospitalized with suicidal ideation. *Am J Psychiatry* 1985;142(5):559-63.
3. Everson SA, Kaplan GA, Goldberg DE, *et al*. Hypertension incidence is predicted by high levels of hopelessness in Finnish men. *Hypertension* 2000;35(2):561-7.
4. Everson SA, Kaplan GA, Goldberg DE, *et al*. Hopelessness and 4-year progression of carotid atherosclerosis. The Kuopio Ischemic Heart Disease Risk Factor Study. *Arterioscler Thromb Vasc Biol 1997;17(8):1490-5.*
5. Whipple MO, Lewis TT, Sutton-Tyrrell K, *et al*. Hopelessness, depressive symptoms, and carotid atherosclerosis in women: the Study of Women's Health Across the Nation (SWAN) heart study. *Stroke* 2009;40(10):3166-72.
6. Dunn SL, Corser W, Stommel M, *et al*. Hopelessness and depression in the early recovery period after hospitalization for acute coronary syndrome. *J Cardiopulm Rehabil* 2006;26(3):152-9.
7. Watson M, Homewood J, Haviland J. Coping response and survival in breast cancer patients: a new analysis. *Stress Health* 2012;28(5):376-80.

8. Molassiotis A, Van Den Akker OB, Milligan DW, *et al*. Symptom distress, coping style and biological variables as predictors of survival after bone marrow transplantation. *J Psychosom Res* 1997;42(3):275-85.
9. Morris T, Pettingale K, Haybittle J. Psychological response to cancer diagnosis and disease outcome in patients with breast cancer and lymphoma. *Psychooncology* 1992;1(2):105-14.
10. Beck AT, Weissman A, Lester D, *et al*. The measurement of pessimism: the hopelessness scale. *J Consult Clin Psychol* 1974;42(6):861-5.
11. Watson M, Haviland JS, Greer S, *et al*. Influence of psychological response on survival in breast cancer: a population-based cohort study. *Lancet* 1999;354(9187):1331-6.
12. Everson SA, Goldberg DE, Kaplan GA, *et al*. Hopelessness and risk of mortality and incidence of myocardial infarction and cancer. *Psychosom Med* 1996;58(2):113-21.
13. Kohout FJ, Berkman LF, Evans DA, *et al*. Two shorter forms of the CES-D (Center for Epidemiological Studies Depression) depression symptoms index. *J Aging Health* 1993;5(2):179-93.
14. Menon U, Gentry-Maharaj A, Hallett R, *et al*. Sensitivity and specificity of multimodal and ultrasound screening for ovarian cancer, and stage distribution of detected cancers: results of the prevalence screen of the UK Collaborative Trial of Ovarian Cancer Screening (UKCTOCS). *Lancet Oncol* 2009;10(4):327-40.
15. Leadbetter S, Hawkins NA, Scholl LE, *et al*. Recruiting women for a study on perceived risk of cancer: influence of survey topic salience and early versus late response. *Prev Chronic Dis* 2013;10:E75.
16. Radloff LS. The CES-D scale a self-report depression scale for research in the general population. *Appl Psychol Meas* 1977;1(3):385-401.
17. Cronbach LJ. Coefficient alpha and the internal structure of tests. *Psychometrika* 1951;16(3):297-334.

For peer review only

18. Streiner DL. Starting at the beginning: an introduction to coefficient alpha and internal consistency. *J Pers Assess* 2003;80(1):99-103.
19. Cicchetti DV. Multiple comparison methods: establishing guidelines for their valid application in neuropsychological research. *J Clin Exp Neuropsychol* 1994;16(1):155-61.
20. Shrout PE, Fleiss JL. Intraclass correlations: uses in assessing rater reliability. *Psychol Bull* 1979;86(2):420-8.
21. Haukoos JS, Lewis RJ. Advanced statistics: bootstrapping confidence intervals for statistics with "difficult" distributions. *Acad Emerg Med* 2005;12(4):360-5.
22. Krueger AB, Schkade DA. The Reliability of Subjective Well-Being Measures. *J Public Econ* 2008;92(8-9):1833-45.
23. Nunnally J. C.(1978). Psychometric theory: New York: McGraw-Hill, 1978.
24. Aiken LR. Psychological testing and assessment. New York: Allyn & Bacon, 2002.
25. Fisher LB, Overholser JC. Refining the Assessment of Hopelessness: An Improved Way to Look to the Future. *Death Stud* 2013;37(3):212-27.
26. Holden RR, Fekken GC. Test-retest reliability of the hopelessness scale and its items in a university population. *J Clin Psychol* 1988;44(1):40-43.
27. Velting DM. Personality and negative expectancies: Trait structure of the Beck Hopelessness Scale. *Pers Individ Dif* 1999;26(5):913-21.
28. Dunn SL, Olamijulo GB, Fuglseth HL, et al. The State-Trait Hopelessness Scale: development and testing. *West J Nurs* Res 2014;36(4):552-70.
29. Jonason PK, Luévano VX. Walking the thin line between efficiency and accuracy: Validity and structural properties of the Dirty Dozen. *Pers Individ Dif* 2013;55(1):76-81.
30. Mitchell AJ, Coyne JC. Do ultra-short screening instruments accurately detect depression in primary care? A pooled analysis and meta-analysis of 22 studies. *Br J Gen Pract* 2007;57(535):144-51.

For peer review only

31. Kruyen PM, Emons WH, Sijtsma K. Shortening the S-STAI: consequences for research and clinical practice. *J Psychosom Res* 2013;75(2):167-72.

32. Beck AT, Steer RA, Ranieri WF. Scale for Suicide Ideation: psychometric properties of a self-report version. *J Clin Psychol* 1988;44(4):499-505.

33. Pompili M. Exploring the phenomenology of suicide. *Suicide Life Threat Behav* 2010;40(3):234-44.

34. Yip PS, Cheung YB. Quick assessment of hopelessness: a cross-sectional study. *Health Qual Life Outcomes* 2006;4(1):13.

35. Löwe B, Wahl I, Rose M, *et al*. A 4-item measure of depression and anxiety: validation and standardization of the Patient Health Questionnaire-4 (PHQ-4) in the general population. *J Affect Disord* 2010;122(1):86-95.

For peer review only

TITLE

Identifying hopelessness in population research: A validation study of two brief measures of hopelessness.

Lindsay Fraser,[1] Matthew Burnell,[1] Laura Currin Salter,[2] Evangelia-Ourania Fourkala,[1] Jatinderpal Kalsi,[1] Andy Ryan,[1] Sue Gessler,[2,1] Yori Gidron,[3] Andrew Steptoe,[4] Usha Menon[1]

Corresponding author

Ms Lindsay Fraser, Gynaecological Cancer Research Centre, Department of Women's Cancer, UCL Institute for Women's Health, Maple House, 149 Tottenham Court Road, London W1T 7DN, United Kingdom

Email: l.fraser@ucl.ac.uk

Tel: +44 020 3447 2114

Fax: +44 020 3447 2129

Author Affiliations

[1]Department of Women's Cancer, UCL Institute for Women's Health and NIHR University College London Hospitals Biomedical Research Centre, London, UK.

[2]University College London Hospitals NHS Foundation Trust, London, UK.

[3]Faculty of Medicine & Pharmacy, Vrije Universiteit Brussel (VUB), Belgium.

[4]Institute of Epidemiology and Health Care, University College London, London, UK.

Key Words: Hopelessness, Depression/epidemiology, Adult, Cohort studies.

Word Count: 3308 (Abstract=289, Manuscript=2,116, References=860, Tables=114).

For peer review only

ABSTRACT

Objective: Hopelessness is an important construct in psychosocial epidemiology, but there is great pressure on the length of questionnaire measures in large scale population and clinical studies. We examined the validity and test-retest reliability of two brief measures of hopelessness, an existing negatively worded 2-item measure of hopelessness (*Brief-H-Neg*) and a positively worded version of the same instrument (*Brief-H-Pos*).

Design: Cohort study.

Setting: Control arm of the UK Collaborative Trial of Ovarian Cancer Screening.

Participants: A non-clinical research-based sample of 5000 postmenopausal women selected from 56512 participants.

Primary and secondary outcome measures: Spearman rank correlation of brief measures of hopelessness with Beck Hopelessness Scale (*BHS*). Spearman rank correlation with Centre for Epidemiological Studies Depression Scale (*CES-D*) and change in mean score on repeat testing.

Methods: Two short hopelessness measures, a negatively worded brief measure of hopelessness (*Brief-H-Neg*) and positively worded brief measure of hopelessness (*Brief-H-Pos*), were administered by postal questionnaire to 5000 women together with the 20-item Beck Hopelessness Scale (*BHS*) and 20-item Centre for Epidemiologic Studies Depression scale (*CES-D*). The *Brief-H-Neg* and *Brief-H-Pos* were re-administered to 500 women after a two week interval.

Results: 2413 postmenopausal women (mean age 68.9 years) completed the questionnaire. The *Brief-H-Neg* and *Brief-H-Pos* correlated 0.93 and 0.87 with the *BHS* after correction for attenuation and their association with the *CES-D* mirrored that seen with the BHS (Spearman rank correlation 0.88 and 0.68 respectively). There was no change in mean scores on the two measures with repeat testing in the 433 women who completed it and test-retest reliability was good (Intraclass correlations *Brief-H-Neg* 0.67 and *Brief-H-Pos* 0.72).

For peer review only

Conclusions: These findings provide support for the validity of the *Brief-H-Neg* and *Brief-H-Pos*. These brief measures are likely to be useful in large population studies assessing hopelessness.

ARTICLE SUMMARY

Article Focus

- Research into the role of hopelessness on morbidity and mortality is hampered by the lack of validated brief measures for use in large scale clinical and population studies.
- This study explored the validity and reliability of an existing brief measure of hopelessness with negatively worded items, along with a newly created measure with positively worded items which may be preferred in some research settings.

Key Messages

- Both the original negatively worded brief instrument and the new positively worded version are valid and reliable instruments for measuring hopelessness.
- While brief measures of hopelessness necessarily sacrifice some level of detail compared to longer measures, they could be useful when there is great pressure on questionnaire length.
- The predictive validity of the original negatively worded measure has previously been demonstrated and this remains to be tested in the positively worded measure.
- Further testing to verify the construct validity of the two brief measures is warranted.

Strengths and Limitations

- The strength of this study is the large sample size.
- Limitations include generalizability of the results beyond older women and the modest response rate.

For peer review only

INTRODUCTION

Hopelessness is the subjective appraisal of negative expectations about the occurrence of highly valued outcomes coupled with the sense that one lacks control over desired events in the future.[1] Hopelessness has been related to the onset and prognosis of mental and physical health outcomes including the development of depression,[1] suicidal ideation,[2] hypertension,[3] subclinical atherosclerosis,[4, 5] adaptation following acute cardiac events[6] and progression of carotid atherosclerosis.[4] In the psycho-oncology literature hopelessness has been found to predict prognosis in various cancers including breast and haematological cancers,[7, 8] although the evidence is not consistent.

Hopelessness has been measured in clinical and population research in a variety of ways including systematic interviews[9] and validated psychometric measures such as the Beck Hopelessness Scale[10] and the Mental Adjustment to Cancer scale.[11] There is great pressure in large scale population studies on questionnaire size due to the volume of clinical and demographic variables that must be collected. Everson et al[12] devised a 2-item measure of hopelessness which has been used in a number of cardiovascular studies.[3, 4, 12] The reliability of this instrument and its relationship with standard measures has not been established. An additional issue concerns the negative valence of the items (e.g. 'The future seems to me to be hopeless and I can't believe that things are changing for the better'). In preliminary work for the large study in which this research is embedded, some respondents found these items upsetting and this has been confirmed by others.[13] We devised a positively worded 2-item version. We compared both brief measures with established measures of hopelessness and depressive symptoms in a large population sample, and assessed their reliability.

METHODS

Participants

5000 participants were selected from 56512 post-menopausal women in the control arm of the UK Collaborative Trial of Ovarian Cancer Screening (UKCTOCS,[14]

For peer review only

ISRCTN22488978, ClinicalTrials.gov number NCT00058032). The mean age of women invited was 69.6 +/- 6.1 years (range: 57-85).

Procedure

A postal questionnaire comprised of measures of hopelessness and depression was sent to 5000 women (Time 1, T1). After a 2-week interval (Time 2, T2) 500 respondents were asked to repeat the *Brief-H-Neg* (n=250) or the *Brief-H-Pos* (n=250) to assess test-retest reliability. Selection of the retest cohort was staggered based on the date of T1 questionnaire return, as early and late responders may differ on levels of hopelessness or depression.[15]

Measures

The *Brief-H-Neg* is a 2-item measure of hopelessness comprised of negatively valenced statements: 'The future seems to me to be hopeless and I can't believe that things are changing for the better'; 'I feel that it is impossible to reach the goals I would like to strive for'.[12] Everson et al selected these from a battery of psychosocial measures used in the Kuopio Ischemic Heart Disease study, defining hopelessness as negative expectancies about oneself and the future. Respondents indicate agreement on a 5-point scale (range: 2-10), higher scores indicate higher hopelessness (Appendix A).

The *Brief-H-Pos* was derived by reversing the tone of the *Brief-H-Neg* statements from negative to positive and reverse scoring: 'The future seems to me to be hopeful and I believe that things are changing for the better'; 'I feel that it is possible to reach the goals I would like to strive for' (Appendix B).

The *BHS* is a validated 20-item true-false measure assessing current levels of hopelessness.[10] Items include pessimistic statements ('There's no use in really trying to get something I want because I probably won't get it') and optimistic ones ('I look forward to the future with hope and enthusiasm'). Pessimistic ratings are summed (range: 0-20), higher scores indicate higher hopelessness.

The Centre for Epidemiological Studies Depression Scale (*CES-D*) is a validated 20-item measure of depressive symptoms.[16] Responses are based on the frequency of

For peer review only

occurrence during the past week using a 4-point scale (range: 0-60), higher scores indicate more frequent symptoms of depression.

Analyses

Internal consistency was based on Coefficient Alpha[17] with alpha cut-off points 0.70-0.79 described as *adequate* and ≥0.80 as high.[18] Stability was evaluated using test-retest reliability based on the Intraclass Correlation Coefficient (ICC) with cut-offs ≤0.40 for *poor*, 0.41-0.59 *fair*, 0.60-0.74 *good*, ≥0.75 excellent.[19] Estimated variances components derived from a one-way random effects model were used to calculate ICC's.[20] The relationship between study measures was assessed using Spearman's rank correlations (CIs were estimated using bootstrapping with 1000 iterations).[21] To estimate the strength of correlations between study measures, a correction for attenuation arising from measurement error was applied: $\rho_{xy} = r_{xy}$*/square root symbol* $(r_{xx}.r_{yy})$,[22, 23] where ρ_{xy} = true correlation between x and y, r_{xy} = observed correlation between x and y, r_{xx} = estimated reliability of x, r_{yy} = estimated reliability of y. We used published test-retest reliability estimates for r_{xx} and r_{yy}: *BHS* 0.69[24] and *CES-D* 0.67.[16] In the absence of published test-retest data for the *Brief-H-Neg*/*Brief-H-Pos*, we used the ICCs reported in this study. Data were analysed using STATA version 12.1.

RESULTS

Sample Characteristics

The questionnaire was returned by 2413 women (48.3%) (T1) (Table 1). Respondents reported significantly higher levels of education than non-respondents, were younger and more likely to be Caucasian (differences were not clinically significant, due to their small magnitude). 115 respondents (4.77%) scored CES-D ≥16/60, a cut-off indicative of clinically significant depressive symptomatology, suggesting this cohort is not unusually depressed.

Table 1 Description of respondents' characteristics

For peer review only

	**Respondents** (N=2413)
**Age** in years (mean +/- SD)	68.9 +/- 5.9 (range: 57-84)
**Ethnicity** n (%)	
White	2376 (98.7)
Black	11 (0.5)
Asian	7 (0.3)
Other	14 (0.6)
Unknown	5 (0.2)
**Education** n (%)	
Higher (University, Professional)	819 (33.9)
Some (O' Level, A' Level, Clerical)	955 (39.6)
None	610 (25.3)
Unknown	29 (1.2)
**Hopelessness** (mean +/- SD)	
Brief-H-Neg	4.42 +/- 2.21 (n=2402)
Brief-H-Pos	4.74 +/- 1.85 (n=2393)
BHS	4.81 +/- 4.49 (n=2400)
**Depression** (mean +/- SD)	
CES-D	12.44 +/- 10.39 (n=2395)

For peer review only

Concurrent Validity

The *Brief-H-Neg* and *Brief-H-Pos* measures correlated well with the *BHS* and mirrored the positive association seen between the *BHS* and the *CES-D* (Table 2).

Table 2 Correlation between measures of hopelessness and depression

	**Brief-H-Neg**	**Brief-H-Pos**	**BHS**

	(n)	(n)	(n)
**BHS**	0.93 (2393)	0.87 (2384)	
**CES-D**	0.88 (2379)	0.68 (2392)	0.87 (2379)

Stability

433/497 (87.1%) women completed the *Brief-H-Neg* (n=221) or *Brief-H-Pos* (n=212) on two occasions. *Brief-H-Neg*, T1 M = 4.64 +/- 1.74 (*n*=248), T2 M = 4.29 +/- 2.39 (*n*=221); *Brief-H-Pos*, T1 M = 4.61 +/- 1.878 (*n*=249), T2 M = 4.57 +/- 1.96 (*n*=212). The short term test-retest reliability of both measures was good: *Brief-H-Neg* ICC = 0.67 (95% CI 3.98-4.49) and *Brief-H-Pos* ICC = 0.72 (95% CI 4.39-4.83).

Reliability

All study measures demonstrated good internal consistency: *Brief-H-Neg* α 0.80, *Brief-H-Pos* α 0.77, *BHS* α 0.89, *CES-D* α 0.90. Alpha for the *Brief-H-Neg* and *Brief-H-Pos* was lower than the longer BHS and CES-D (alpha is known to rise as the number of items increase).

DISCUSSION

A brief measure is needed to examine the role of hopelessness on mental and physical health outcomes in large population studies. We examined the validity and reliability of two brief measures of hopelessness in a large non-clinical sample, one negatively valenced (*Brief-H-Neg*) and one positively valenced (*Brief-H-Pos*). Both were shown to correlate strongly with the longer BHS and mirror the positive correlation seen between the *BHS* and a measure of depression, providing evidence of concurrent validity, with adequate internal consistency and test-retest reliability.

The size of the 2-week retest correlations for the brief measures reported in our non-clinical sample (0.67 and 0.72) are similar to those reported for the BHS in a sample of

For peer review only

university undergraduates over a 3-week retest interval (0.67, female students) or a 10-week interval (0.75).[25, 26] Studies assessing retest reliability of hopelessness instruments have reported varying retest intervals. Hopelessness may be conceptualised as a temporary mood state reflecting a person's response to challenging circumstances, or a more enduring trait reflecting a habitual outlook on many aspects of life.[27] Most commonly used measures of hopelessness, including the BHS, do not distinguish between state and trait hopelessness. If hopelessness is an enduring trait, measures of hopelessness would be expected to have high test-retest reliability. A measure that does addresses the state versus trait distinction, the State-Trait Hopelessness Scale, has reported retest correlations of state and trait hopelessness over a 6-week interval (state 0.65, trait 0.74) and over a 6-month interval (state 0.61, trait 0.78) in hospitalised patients with coronary heart disease.[28] Again, the size of these retest correlations are not dissimilar to those seen in the brief measures reported in our study after a 2-week interval.

The selection of a measure is determined to an extent by the practical context of the investigation. Very brief measures necessarily sacrifice some level of detail compared with their longer counterparts.[29] A pooled analysis and meta-analysis of 22 studies involving ultra-short (one-, two-, three- or four-item) tests concluded that 2-item and 3-item measures of depression identify 8 out of 10 cases in primary care settings, albeit at the expense of a high false positive rate.[30] This makes them inappropriate diagnostic tests for clinical decision making, but suitable as screening tools in primary care as well as in population cohort research where participants have to complete a number of demographic and clinical questions in addition to psychological measures.[31]

Our data suggest that while 2-item measures of hopelessness may not have the detail of the 20-item BHS measure, they do have adequate reliability to be used in large population based studies. The reduced burden on participants may encourage a high response rate. The 5-point Likert response scales of the *Brief-H-Neg* and *Brief-H-Pos* provide a reasonable range of scores to work with. However, if information on the hypothesised affective, motivational and cognitive aspects of hopelessness is required in

For peer review only

order for example to target a therapeutic intervention, the 20-item BHS would be more suitable, because a total score for each dimension can be derived from the summed individual items of the scale.[10]

The results of this study provide preliminary support for the construct validity of both brief measures of hopelessness but further testing of their construct validity is required, along with tests of their predictive validity on physical and mental health outcomes. It would be helpful to examine the psychometric properties of both brief measures in a psychiatric sample where higher levels of hopelessness are expected, such as a group of hospitalised patients who have attempted suicide.[32] There is good evidence that hopelessness is associated with suicidal ideation and is recognised as a better predictor for suicidal intent than depression.[33] Moreover, brief measures of hopelessness derived from the BHS including a 4-item scale and to a lesser extent a single item, have been shown to perform as well as the 20-item BHS in identifying people with suicidal ideation.[34] The predictive validity for the *Brief-H-Neg* on physical health outcomes has been shown in studies exploring the relationship between hopelessness and disease incidence and mortality, and this remains to be addressed for the *Brief-H-Pos*.[3-5, 12]

There are some limitations to this study. Firstly, the sample of older women limits the generalizability of the results. It would be useful to validate the *Brief-H-Neg* and *Brief-H-Pos* in a general population sample and to generate normative data, as has been shown for 2-item measures of depression (PHQ-2) and anxiety (GAD-2).[35] Secondly, the response rate of 48.3% is modest, although importantly there was no evidence of bias between responders and non-responders and the sample of responders is large. It is perhaps unsurprising that many of the women invited from the control arm of an ovarian cancer screening study were not motivated to take part in this nested study assessing brief measures of hopelessness. Lastly, we did not directly test the assumption that those suffering from low mood may find it difficult to be confronted with the negatively phrased questions of the *Brief-H-Neg* compared with the positively phrased *Brief-H-Pos*.

For peer review only

CONCLUSION

Both Everson et al's negatively valenced measure of hopelessness (*Brief-H-Neg*) and the positively valenced measure (*Brief-H-Pos*) developed as a potentially less stressful measure for participants in health research have been shown to be valid and reliable measures of hopelessness. Further testing to verify their construct validity is warranted. Meanwhile the findings suggest that these brief measures are fit for purpose in large scale population studies investigating the association of hopelessness and health outcomes. Evidence of a consistent association with mortality in such studies would add impetus to the search for interventions that can modify the risk.

Contributors

All authors contributed to the study design. JK and AR collected the data. MB conducted the data analysis. MB, LF, UM and AS interpreted the data. LF, UM and AS drafted the manuscript. All authors revised and approved the final version of the manuscript.

Funding

UKCTOCS is core funded by the Medical Research Council (G0801228), Cancer Research UK (C1479/A2884), The Department of Health, with additional support from The Eve Appeal, Special Trustees of Barts and the London, Special Trustees of UCLH. The funding bodies had no role in study design, data collection, analysis, interpretation or writing of this report.

Competing Interests

The authors have no competing interests to report.

Patient Consent

Obtained.

Ethics Approval

Ethical approval was obtained from the NRES Committee North West-Haydock (00/8/034).

Provenance and Peer Review

Not commissioned; externally peer reviewed.

Data Sharing Agreement

For peer review only

The data from the current study are available to specific researchers at the Gynaecological Cancer Research Centre, UCL Institute for Women's Health.

Open Access

This is an Open Access article distributed in accordance with the Creative Commons Attribution Non Commercial (CC BY-NC 3.0) license, which permits others to distribute, remix, adapt, build upon this work non-commercially, and license their derivative works on different terms, provided the original work is properly cited and the use is non-commercial. See: http://creativecommons.org/licenses/by-nc/3.0/

REFERENCES

1. Alloy LB, Abramson LY, Metalsky GI, *et al*. The hopelessness theory of depression: attributional aspects. *Br J Clin Psychol* 1988;27(Pt 1):5-21.
2. Beck AT, Steer RA, Kovacs M, *et al*. Hopelessness and eventual suicide: a 10-year prospective study of patients hospitalized with suicidal ideation. *Am J Psychiatry* 1985;142(5):559-63.
3. Everson SA, Kaplan GA, Goldberg DE, *et al*. Hypertension incidence is predicted by high levels of hopelessness in Finnish men. *Hypertension* 2000;35(2):561-7.
4. Everson SA, Kaplan GA, Goldberg DE, *et al*. Hopelessness and 4-year progression of carotid atherosclerosis. The Kuopio Ischemic Heart Disease Risk Factor Study. *Arterioscler Thromb Vasc Biol 1997;17(8):1490-5.*
5. Whipple MO, Lewis TT, Sutton-Tyrrell K, *et al*. Hopelessness, depressive symptoms, and carotid atherosclerosis in women: the Study of Women's Health Across the Nation (SWAN) heart study. *Stroke* 2009;40(10):3166-72.
6. Dunn SL, Corser W, Stommel M, *et al*. Hopelessness and depression in the early recovery period after hospitalization for acute coronary syndrome. *J Cardiopulm Rehabil* 2006;26(3):152-9.
7. Watson M, Homewood J, Haviland J. Coping response and survival in breast cancer patients: a new analysis. *Stress Health* 2012;28(5):376-80.

For peer review only

8. Molassiotis A, Van Den Akker OB, Milligan DW, *et al*. Symptom distress, coping style and biological variables as predictors of survival after bone marrow transplantation. *J Psychosom Res* 1997;42(3):275-85.

9. Morris T, Pettingale K, Haybittle J. Psychological response to cancer diagnosis and disease outcome in patients with breast cancer and lymphoma. *Psychooncology* 1992;1(2):105-14.

10. Beck AT, Weissman A, Lester D, *et al*. The measurement of pessimism: the hopelessness scale. *J Consult Clin Psychol* 1974;42(6):861-5.

11. Watson M, Haviland JS, Greer S, *et al*. Influence of psychological response on survival in breast cancer: a population-based cohort study. *Lancet* 1999;354(9187):1331-6.

12. Everson SA, Goldberg DE, Kaplan GA, *et al*. Hopelessness and risk of mortality and incidence of myocardial infarction and cancer. *Psychosom Med* 1996;58(2):113-21.

13. Kohout FJ, Berkman LF, Evans DA, *et al*. Two shorter forms of the CES-D (Center for Epidemiological Studies Depression) depression symptoms index. *J Aging Health* 1993;5(2):179-93.

14. Menon U, Gentry-Maharaj A, Hallett R, *et al*. Sensitivity and specificity of multimodal and ultrasound screening for ovarian cancer, and stage distribution of detected cancers: results of the prevalence screen of the UK Collaborative Trial of Ovarian Cancer Screening (UKCTOCS). *Lancet Oncol* 2009;10(4):327-40.

15. Leadbetter S, Hawkins NA, Scholl LE, *et al*. Recruiting women for a study on perceived risk of cancer: influence of survey topic salience and early versus late response. *Prev Chronic Dis* 2013;10:E75.

16. Radloff LS. The CES-D scale a self-report depression scale for research in the general population. *Appl Psychol Meas* 1977;1(3):385-401.

17. Cronbach LJ. Coefficient alpha and the internal structure of tests. *Psychometrika* 1951;16(3):297-334.

For peer review only

18. Streiner DL. Starting at the beginning: an introduction to coefficient alpha and internal consistency. *J Pers Assess* 2003;80(1):99-103.

19. Cicchetti DV. Multiple comparison methods: establishing guidelines for their valid application in neuropsychological research. *J Clin Exp Neuropsychol* 1994;16(1):155-61.

20. Shrout PE, Fleiss JL. Intraclass correlations: uses in assessing rater reliability. *Psychol Bull* 1979;86(2):420-8.

21. Haukoos JS, Lewis RJ. Advanced statistics: bootstrapping confidence intervals for statistics with "difficult" distributions. *Acad Emerg Med* 2005;12(4):360-5.

22. Krueger AB, Schkade DA. The Reliability of Subjective Well-Being Measures. *J Public Econ* 2008;92(8-9):1833-45.

23. Nunnally J. C.(1978). Psychometric theory: New York: McGraw-Hill, 1978.

24. Aiken LR. Psychological testing and assessment. New York: Allyn & Bacon, 2002.

25. Fisher LB, Overholser JC. Refining the Assessment of Hopelessness: An Improved Way to Look to the Future. *Death Stud* 2013;37(3):212-27.

26. Holden RR, Fekken GC. Test-retest reliability of the hopelessness scale and its items in a university population. *J Clin Psychol* 1988;44(1):40-43.

27. Velting DM. Personality and negative expectancies: Trait structure of the Beck Hopelessness Scale. *Pers Individ Dif* 1999;26(5):913-21.

28. Dunn SL, Olamijulo GB, Fuglseth HL, et al. The State-Trait Hopelessness Scale: development and testing*. West J Nurs* Res 2014;36(4):552-70.

29. Jonason PK, Luévano VX. Walking the thin line between efficiency and accuracy: Validity and structural properties of the Dirty Dozen. *Pers Individ Dif* 2013;55(1):76-81.

30. Mitchell AJ, Coyne JC. Do ultra-short screening instruments accurately detect depression in primary care? A pooled analysis and meta-analysis of 22 studies. *Br J Gen Pract* 2007;57(535):144-51.

For peer review only

31. Kruyen PM, Emons WH, Sijtsma K. Shortening the S-STAI: consequences for research and clinical practice. *J Psychosom Res* 2013;75(2):167-72.

32. Beck AT, Steer RA, Ranieri WF. Scale for Suicide Ideation: psychometric properties of a self-report version. *J Clin Psychol* 1988;44(4):499-505.

33. Pompili M. Exploring the phenomenology of suicide. *Suicide Life Threat Behav* 2010;40(3):234-44.

34. Yip PS, Cheung YB. Quick assessment of hopelessness: a cross-sectional study. *Health Qual Life Outcomes* 2006;4(1):13.

35. Löwe B, Wahl I, Rose M, *et al*. A 4-item measure of depression and anxiety: validation and standardization of the Patient Health Questionnaire-4 (PHQ-4) in the general population. *J Affect Disord* 2010;122(1):86-95.

For peer review only

Appendix A

Figure 1 *Brief-Neg-H* Measure

For each of the statements below, please tick the box that best applies to you.

1. The future seems to me to be hopeless and I can't believe that things are changing for the better

☐ 5	☐ 4	☐ 3	☐ 2	☐ 1
absolutely agree	somewhat agree	cannot say	somewhat disagree	absolutely disagree

2. I feel that it is impossible to reach the goals I would like to strive for

☐ 5	☐ 4	☐ 3	☐ 2	☐ 1
absolutely agree	somewhat agree	cannot say	somewhat disagree	absolutely disagree

Scores are shown next to each item response; total score range 2-10 (higher scores indicate higher hopelessness).

Appendix B

Figure 2 *Brief-H-Pos* Measure

For each of the statements below, please tick the box that best applies to you.

1. The future seems to me to be hopeful and I believe that things are changing for the better

☐ 1	☐ 2	☐ 3	☐ 4	☐ 5
absolutely agree	somewhat agree	cannot say	somewhat disagree	absolutely disagree

2. I feel that it is possible to reach the goals I would like to strive for

☐ 1	☐ 2	☐ 3	☐ 4	☐ 5
absolutely agree	somewhat agree	cannot say	somewhat disagree	absolutely disagree

Scores are shown next to each item response and reverse scored; total score range 2-10 (higher scores indicate higher hopelessness).
